# Human Wharton’s Jelly-Derived Mesenchymal Stromal Cells Primed by Tumor Necrosis Factor-α and Interferon-γ Modulate the Innate and Adaptive Immune Cells of Type 1 Diabetic Patients

**DOI:** 10.3389/fimmu.2021.732549

**Published:** 2021-09-28

**Authors:** Mairvat Al- Mrahleh, Suzan Matar, Hanan Jafar, Suha Wehaibi, Nazneen Aslam, Abdalla Awidi

**Affiliations:** ^1^ Cell Therapy Center, The University of Jordan, Amman, Jordan; ^2^ Department of Clinical Laboratory Science, The University of Jordan, School of Science, Amman, Jordan; ^3^ Department of Anatomy & Histology, The University of Jordan, School of Medicine, Amman, Jordan; ^4^ Department of Hematology & Oncology, The University of Jordan, School of Medicine, Amman, Jordan

**Keywords:** type 1 diabetes, Immunomodulation, priming, Wharton’s jelly-derived mesenchymal stromal cells, regulatory T cells, tolerogenic dendritic cells

## Abstract

The unique immunomodulation and immunosuppressive potential of Wharton’s jelly-derived mesenchymal stromal cells (WJ-MSCs) make them a promising therapeutic approach for autoimmune diseases including type 1 diabetes (T1D). The immunomodulatory effect of MSCs is exerted either by cell-cell contact or by secretome secretion. Cell-cell contact is a critical mechanism by which MSCs regulate immune-responses and generate immune regulatory cells such as tolerogenic dendritic cells (tolDCs) and regulatory T cell (Tregs). In this study, we primed WJ-MSCs with TNF-α and IFN-γ and investigated the immunomodulatory properties of primed WJ-MSCs on mature dendritic cells (mDCs) and activated T cells differentiated from mononuclear cells (MNCs) of T1D patient’s. Our findings revealed that primed WJ-MSCs impaired the antigen-mediated immunity, upregulated immune-tolerance genes and downregulated immune-response genes. We also found an increase in the production of anti-inflammatory cytokines and suppression of the production of pro-inflammatory cytokines. Significant upregulation of *FOXP3, IL10* and *TGFB1* augmented an immunosuppressive effect on adaptive T cell immunity which represented a strong evidence in support of the formation of Tregs. Furthermore, upregulation of many critical genes involved in the immune-tolerance mechanism (*IDO1* and *PTGES2/PTGS)* was detected. Interestingly, upregulation of *ENTPD1/NT5E* genes express a strong evidence to switch immunostimulatory response toward immunoregulatory response. We conclude that WJ-MSCs primed by TNF-α and IFN-γ may represent a promising tool to treat the autoimmune disorders and can provide a new evidence to consider MSCs- based therapeutic approach for the treatment of TID.

## Introduction

Type 1 diabetes (T1D) is a T-cell mediated autoimmune disorder in which insulin-secreting β-cells of the pancreas are selectively destroyed. The mechanisms involved in the β-cell destruction are still not understood. Currently, tyrosine kinase, islet-specific glucose-6-phosphatase catalytic subunit-related protein (IGRP), insulinoma-associated protein 2 (IA-2), zinc transporter (ZnT), and glutamic acid decarboxylase 65 (GAD65) are all considered to be involved in the β-cell specific autoimmune process (1, 2). However, GAD65 is identified as the major antigen targeted by T-cell autoantibodies in T1D (3).

Mature dendritic cells (mDCs) and T cells are implicated in the pathogenesis of T1D. Mature dendritic cells play a key role in all stages of β-cells destruction due to their immunostimulatory effect on naïve T cells (4). As a result, mDCs trigger two important functions in controlling T-cell immunity; T-cell activation by the expression of T-cell antigen and secretion of specific cytokines which determine the nature of T-cell responses (5).

To date, the most reliable approach for the management of T1D remains the islet or pancreas transplantation but there are many obstacles in their use that include; allo-immune graft rejection, recurrence of autoimmunity, as well as inadequate supply of donor tissues (6–8). Hence, there is a need for an efficacious alternative approach to control T1D.

In the past few decades, human mesenchymal stromal cells (MSCs) presented a promising strategy for treating various immune-mediated diseases including T1D due to their ability to interact with many types of immune cells. Furthermore, MSCs exert immunomodulatory and anti-inflammatory effects on the adaptive and innate immune system both *in vivo* and *in vitro* (9, 10), through their secretion of chemokines, cytokines, growth factors, and biological active substances (11, 12). Unfortunately, many limitations have threatened the use of MSCs-based therapies such as, cell senescence, loss of function after cryopreservation, and unpredictability of MSCs behavior *in vivo* (13, 14). On the other hand, various studies demonstrated that under normal conditions, MSCs express low or insufficient levels of immunomodulatory and immunosuppressive factors (15–17). For these reasons, many ongoing researches proposed the requirement of a practical approach to improve MSCs survival, differentiation potential, therapeutic efficacy, and immunomodulatory functions (18–20). Recently, cell priming or cell activation is considered one of the most interesting functional approaches (21–23). “Human mesenchymal stromal cells Priming” or “MSCs licensing” is defined as the exposure of MSCs to pro-inflammatory cytokines, such as IFN-*γ*, TNF-*α*, IL-1*α* and IL-1*β*, to increase their anti-inflammatory and immunomodulatory effects on the innate and adaptive immunity (24, 25).

Human mesenchymal stromal cells can strongly interact with many types of immune cells either by cell-cell contact or by their secretome (26). Cell-cell contact is a critical immunosuppressive mechanism of MSCs due to their ability to express a wide range of chemokine receptors and surface adhesion molecules, such as CXCR3, CCR5, CXCL9, CXCL10, CXCL11, VCAM, ICAM-1 and ICAM-2, which enhance their affinity to bind to immune cells and exert their immunomodulatory functions (27–31).

We hypothesized that preconditioning Wharton’s jelly-derived mesenchymal stromal cells (WJ-MSCs) with IFN-*γ* and TNF-*α* may lead to robust immunomodulatory effects on immune cells leading to the induction of immunoregulatory cells such as tolDCs and Tregs. Wharton’s jelly-derived mesenchymal stromal cells are optimal candidates for cellular therapies in allogeneic transplantation due to their low immunogenicity and their immunomodulatory properties in addition to their ability to release large amounts of tolerogenic factors by direct and indirect contact with immune cells. Therefore, this study aimed to investigate the effect of primed WJ-MSCs on the profile and functions of mDCs and activated T cells that differentiated from T1D patients.

## Materials and Methods

### Human Platelet Lysate Preparation

According to Awidi et al. (32), human platelet lysate (PL) was obtained from different platelet apheresis collections at blood banking unit in the Jordan University Hospital, Jordan. The count of platelets was performed using automated hematology analyzer. Pooled samples were subjected to three repeated temperature cycles, frozen at -80°C, then heated at 37°C, then frozen and stored at - 20°C until experimental use.

### Isolation and Culture of WJ-MSCs

Human WJ-MSCs were obtained and processed immediately after cesarean section (n=5). All donors signed a consent form before delivery. The protocol was approved by Institutional Review Board (IRB) committee of the Cell Therapy Center, Jordan. (IRB NO. 07-11-2019). Briefly, MSCs were isolated from the Wharton’s Jelly region of the Umbilical Cord (UC). The cords were washed twice with PBS supplemented with 100u/ml Penicillin-Streptomycin, cut into 2 mm^2^ pieces and placed into 148 cm^2^ tissue culture plates containing α-MEM supplemented with 10% PL, 100u/ml penicillin/streptomycin and 2mM/ml L-Glutamine. Plates were incubated in a 5% CO2 incubator at 37°c and 95% relative humidity for 7 to 9 days. The medium was changed every 2–3 days. After that, the pieces were removed and the cells were harvested and seeded into T-175 flask for 4 passages. For all experiments, MSCs were used at passages 3-4.

To induce primed cells, WJ-MSCs were cultured in 6- well plates (1x10^5/well) and stimulated with recombinant human 50ng/ml TNF- *α* and 50ng/ml IFN-*γ* (R&D Systems, USA) for 48 hours. then cells were washed and stromal cell markers were characterized using hMSCs Analysis kit (BD, UK). The differentiation potential was determined by using StemPro^®^ Differentiation Kit (GIBCO, USA). In parallel experiment, WJ-MSCs were cultured in 6- well plates (1x10^5/well) for 48 hours, then the morphological features of unprimed and primed WJ-MSCs were assessed using Evos Cell Imaging System AMEX1200 (Life Technologies, USA) and the morphological differences were analyzed by Imagej software. The concentration of stimulants was chosen depending on WJ-MSCs morphological changes and based on the expression of immunomodulatory factors.

### Generation and Culture of Human mDCs

All patients signed a consent form before blood samples collection. The data of patients groupes were documented before samples collection (**Table 1**). Fresh heparinized peripheral blood was obtained from five newly onset T1D patients recruited from the Jordan University Hospital, Jordan. In short, mononuclear cells (MNCs) were separated by a Ficoll-Paque gradient centrifugation (specific gravity, 1.077g/ml- Sigma-Aldrich, UK) and cultured in 6-well plates with RPMI 1640 supplemented with 10% PL, 100u/ml penicillin/streptomycin and 2Mm/ml L-Glutamine for two hours in a 5% CO2 incubator at 37°c and 95% relative humidity. Non-adherent cells, T lymphocytes, were collected and tested for CD3+ expression (86.9 ± 5.9) by Flow cytometry using CD3-APC (Biolegend, USA). Adherent cells, monocytes, were washed twice with PBS and were then evaluated for CD14+ expression (92.3± 2) by flow cytometry using CD14-FITC (BD Bioscience, USA). Monocytes were differentiated into immature DCs (iDCs) using 100 ng/ml granulocyte macrophage-colony stimulating factor (GM-CSF) and 50 ng/ml IL-4 (R&D Systems, USA) and incubated for 5 days. Immature DCs were pulsed at a concentration of 1x10^6^ cells/ml with 10µg/ml GAD65 (Abcam, UK), and cultured with 50 ng/ml IL-1*β* and 50 ng/ml TNF- *α* (R&D Systems, USA) for 48 hours to generate mDCs. The concentrations were chosen according to Favaro et al. (33). All cells were cultured in 6-well plates with RPMI 1640 supplemented with 10% PL, 100u/ml penicillin/streptomycin and 2Mm/ml L-Glutamine.

**Table 1:** Data of the study groups of T1D patients.

Patient no	Gender	Age	C-Peptide (NR 1.1-4.4ng/ml)	GAD65	HbA1c (NR 4.8%-5.8%)
1	Male	34	0.83 ng/ml	Positive	6.4%
2	Male	18	0.56 ng/ml	Positive	8.1%
3	Female	21	0.79 ng/ml	Positive	7.4%
4	Male	17	2.8 ng/ml	Positive	9.5%
5	Female	22	1.1 ng/ml	Positive	5.2%

NR, Normal Range.

### T Cells Activation and Proliferation Assay

T lymphocytes were stained with carboxyfluorescein succinimidyl ester (CFSE). The concentration of CFSE fluorescent dye is reduced by cell division. During cell division, CFSE is distributed among daughter cells. Hence, proliferation is assessed by dilution of the CFSE dye (34). Briefly, 1x10^6^ lymphocytes were incubated with CFSE (10mM; Abcam, UK) for 10 minutes in a 5% CO2 incubator at 37°c and 95% relative humidity. The reaction was stopped with RPMI supplemented with 10%PL. T Lymphocytes were co-cultured with GAD65 pulsed mDCs (mDCs: T cell ratio, 1:10) in 6-well plates with RPMI 1640 supplemented with 10% PL, 100u/ml penicillin/streptomycin, 2Mm/ml L-Glutamine, and pulsed at concentration of 1x10^6^ cells/ml with 10µg/ml GAD65 (Abcam, UK) for 4 days (referred to as activated T cells). After that, activated T cells (CD4+ and CD8+) were tested using flow cytometry. In a parallel experiment, T lymphocytes were cultured alone under the same conditions without activation (referred to as inactivated T cells).

### WJ-MSCs Co-Cultured With GAD65 Pulsed mDCs and T Cells

Primed WJ-MSCs were cultured overnight in 6-well plates (1x10^5 cells/well) with α-MEM media (Gibco, UK) supplemented with 10% platelet lysate (PL), 100u/ml penicillin/streptomycin and 2Mm/ml L-Glutamine. The medium was replaced by RPMI 1640 supplemented with 10% PL, 100u/ml penicillin/streptomycin and 2Mm/ml L-Glutamine. After that, GAD65-pulsed mDCs were co-cultured with primed WJ-MSCs monolayer (mDCs: WJ-MSCs, ratio 1:1) for 48hours. The ratio was chosen according to Favaro et al. (33). In parallel experiment, primed WJ-MSCs were cultured overnight in 6-well plates (1x10^5 cells/well) with α-MEM media (Gibco, UK) supplemented with 10% platelet lysate (PL), 100u/ml penicillin/streptomycin and 2Mm/ml L-Glutamine. The medium was replaced with RPMI 1640 supplemented with 10% PL, 100u/ml penicillin/streptomycin and 2Mm/ml L-Glutamine. Then, activated T cells were co-cultured with primed WJ-MSCs monolayer (WJ-MSCs: T cells, ratio 1:5) for 48hours. The ratio was chosen according to Liu et al. (35).

### Flow Cytometry Analysis of DCs and T Cells

The expression of co-stimulatory molecules and maturation surface markers protein of iDCs, mDCs and mDCs co-cultured with primed WJ-MSCs were analysed by flow cytometer using the following antibodies: CD14- PE-CY7, CD83- PE-CY5, CD80-FITC, CD86- BV421, CD1a-PE, CD40-BV510, CD209-APC and HLA-DR- Percp-CY5.5, all antibodies are purchased from (BD Bioscience, USA). Cell proliferation and cell-surface marker expression of activated T cells (CD4+ and CD8+) were detected using the following antibodies: CD4-APC and CD8- PE-CY7 (Biolegend, USA). Activation of T cells was detected using CD69-PE (Biolegend, USA).

### Enzyme- Linked Immunosorbent Spot (ELISPOT)

T lymphocytes (2x 10^5) were pulsed with GAD65 and were then cultured under the following conditions: inactivated T cells, activated T cells cultured with or without primed WJ-MSCs. IFN-*γ* ELISPOT analysis was performed as previously described (36, 37) and according to the manufacturer’s instructions (Abcam, UK). All patients were positive for GAD65 antigen.

### Cytokine Quantification by ELISA

Levels of secreted IL-10, IL-6, and TGF-*β* in mDCs were measured in cell-free culture supernatant under the following conditions: primed WJ-MSCs alone, mDCs alone, and primed WJ-MSCs co-cultured with mDCs. Regarding T cells cytokines, levels of secreted IL-10, IL-6, IFN-*γ*, IL-17 and TGF-*β* were measured in cell-free culture supernatant under the following conditions: inactivated T cells alone, activated T cells alone and primed WJ-MSCs co-cultured with activated T cells. All cytokine kits were purchased from Abcam, UK.

### qPCR for Quantification of Gene Expression

To determine the expression of the target genes at the mRNA level, qPCR was performed. Shortly, mDCs and activated T cells were collected from culture by gentle pipetting followed by centrifugation, then the cell pellets were lysed by Trizol-hybrid method for RNA extraction using RNeasy micro kit (Qiagen, USA). The extracted RNA was quantified by a Nanodrop (Thermofisher, USA). To synthesize cDNA, 0.5 μg of total RNA was reverse transcribed by using the PrimeScript RT Master Mix (Takara, China) using T100™ Thermal cycler PCR instrument (BioRad, USA). Primers were designed using Primer-BLAST (RRID : SCR- 003095) and obtained from IDT (USA) (**Table 2**). The qPCR reaction mix was prepared by mixing 25 ng of cDNA with and 200 nM of gene-specific forward and reverse primers (IDT), 7.2 μL of free nuclease water, and10 μL of SYBR Premix Ex Taq II (Tli RNaseH Plus) (Takara, China). The amplification was performed on CFX96 C1000 Touch thermal cycler (BioRad, USA) with the following temperature setting: (i) 95°C for 3 minutes, (ii) 40 cycles 95°C for 5 seconds and 61°C for 30 seconds. 18s rRNA was used as a reference gene. Each sample was performed in triplicate, and a mean value was calculated. Data were analyzed according to 2−ΔΔCT method using CFX Maestro™ Software - Bio-Rad.

**Table 2:** qPCR primer sequences of immunostimulatory and immunomodulatory genes that are involved in the immunoregulatory effect of primed WJ-MSCs.

Gene	Forward Primers Sequence	Reverse Primers Sequence
IL10	5′ GCTGAGAACCAAGACCCAGA3′	5′ AAGAAATCGATGACAGCGCC3′
IL6	5′GGCACTGGCAGAAAACAACC3′	5′GCAAGTCTCCTCATTGAATCC3′
TGFB1	5′GGAAATTGAGGGCTTTCGCC3′	5′CCGGTAGTGAACCCGTTGAT3′
IL17A	5′CGGACTGTGATGGTCAACCT3′	5′TCCTCATTGCGGTGGAGATT3′
TNFA	5′CCTGTAGCCCATGTTGTAGCAAA3′	5′TTATCTCTCAGCTCCACGCCA3′
IFNG	5′GAGTGTGGAGACCATCAAGGA3′	5′TGGACATTCAAGTCAGTTACCGAA3′
FOXP3	5′GAACCTTCCAGGGCCGAGAT3′	5′ATGGTGGCATGGGGTTCAAG3′
IL2	5′TTTTACATGCCCAAGAAGGCCA3′	5′TCCTCCAGAGGTTTGAGTTCT3′
PTGES2	5′GGTGCCTGGTCTGATGATGT3′	5′GATTAGCCTGCTTGTCTGGAAC3′
IDO1	5′CTCTGCCAAATCCACAGGAAA3′	5′CAACTCTTTCTCGAAGCTGGC3′
NT5E	5′CGCTCAGAAAGTGAGGGGTG3′	5′GGAAGGTGGATTGCCTGTGTA3′
ENTPD1	5′CTCAGCCTTGGGAGGAGATAA3′	5′ATGTGCTCCCAGGAATCAGC3′
PTGS1	5′AGCCCTTCAATGAGTACCGC3′	5′TGCCATCTCCTTCTCTCCTAC3′
18S rRNA	5′CGGCGACGACCCATTCGAAC3′	5′GAATCGAACCCTGATTCCCCGTC3′

### Statistical Analysis

Kolmogorov-Smirnov test was used to verified the normal distribution of data. Graph Pad Prism 6 was used for statistical analysis. Data were presented as Mean ± Standard Deviation (SD). One-way ANOVA, unpaired *t*-test, and two-way ANOVA with Tukey’s *post hoc* analysis were performed to analyze the differences between experimental points. qPCR data were analyzed according to 2−ΔΔCT method using CFX Maestro™ Software - Bio-Rad. In all analyses P values <0.05 were considered significant. In qPCR Data Analyses P values <0.01 were considered significant. The used test was described in the figure legends.

## Results

### Characterization and Differentiation of Primed WJ-MSCs

**Figure 1:**
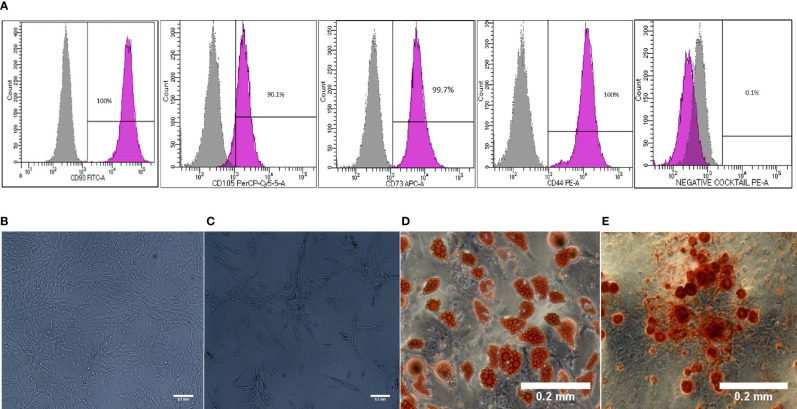
Characterization and differentiation potential of WJ-MSCs priming with IFN-γ and TNF-α **(A)** Representative flow cytometry histograms showing high expression of CD90, CD105, CD73 and CD44 and lack the expression of negative MSCs cocktail (CD34, CD11b, CD19, CD45 and HLA-DR). Gray peak corresponds with isotype control and the violet peak corresponds with the antibodies. **(B)** Unprimed WJ-MSCs. **(C)** Primed WJ-MSCs. **(D)** Oil red staining of primed WJ-MSCs after culture in adipogenic differentiation media. **(E)** Alizarin red staining of primed WJ-MSCs after culture with Osteogenic differentiation media. Data were calculated from five samples. Experiments performed in triplicate for each sample. Data were analyzed using FACS canto II. Magnification = 100x.

Morphology and surface marker expression of WJ-MSCs were evaluated at passage 3. Primed WJ-MSCs expressed typical MSCs surface markers CD90 (100% ± 0.0%), CD73 (99.7% ± 0.2%), CD44 (100% ± 0.0%), and CD105 (90.1% ± 4%), and did not express hematopoietic markers; CD34, CD11b, CD19, CD45 and HLA-DR (0.1% ± 0.05%) (**Figure 1A**). Primed WJ-MSCs exhibited unique morphological features after their priming with TNF- α and IFN-γ compared with unprimed WJ-MSCs (**Figures 1B, C** and **Table 3**). The change of MSCs morphology upon TNF- α and IFN-γ stimulation strongly correlated with a previous study that investigated the morphological features of bone marrow derived MSCs (BM-MSCs after being primed with IFN-*γ* (38).

**Table 3:** Morphological features of WJ-MSCs before and after priming with IFN-γ and TNF-α.

WJ-MSCs morphological features	Unprimed WJ-MSCs	Primed WJ-MSCs
Irregularity	Decrease	Increase
Range	Increase	Decrease
Circumferences	Decrease	Increase
Form factor	Increase	Decrease
Eccentricity	Decrease	increase
Rigidity	Increase	Decrease
Aspect ratio	Decrease	Increase
Nucleus-cytoplasm ratio	Increase	Decrease
Morphological response	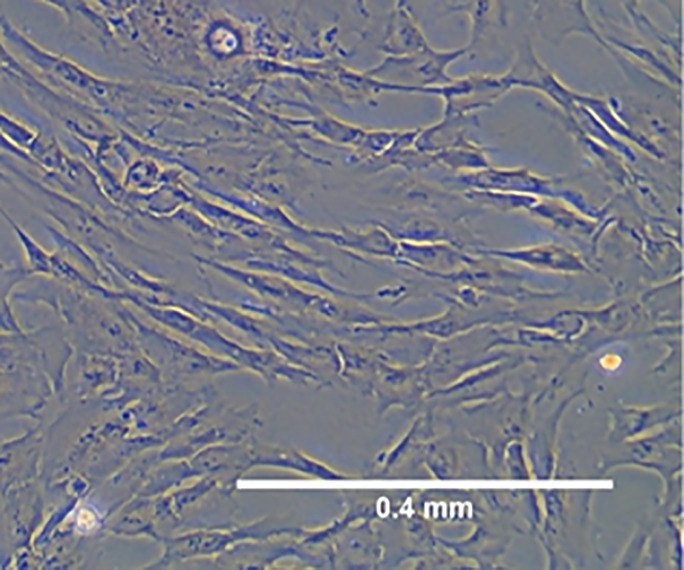	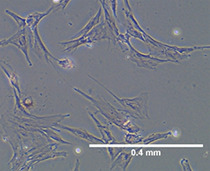

Morphological features were investigated using Evos Cell Imaging System at 20X magnification and the morphological variations were analyzed by ImageJ software.

Primed WJ-MSCs were grown in adipogenic and osteogenic induction media for 14-21 days. All primed WJ-MSCs demonstrated the multilineage differentiation potential (**Figures 1D, E**).

### Effect of Primed WJ-MSCs on GAD65 Pulsing mDCs Profile and Function

**Figure 2:**
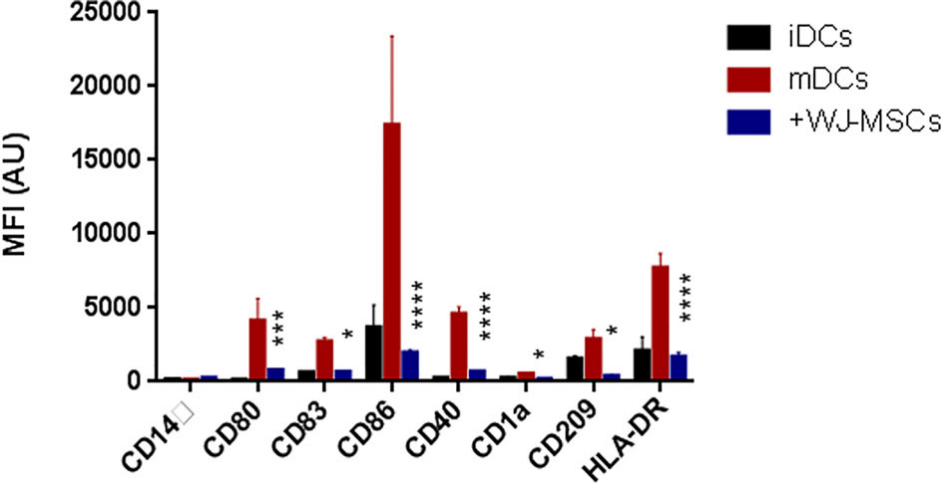
Phenotype of DCs. Mean fluorescence intensity (MFI) ± SD for the surface intensity of co-stimulatory molecules and maturation markers on iDCs (black), GAD65 pulsed mDCs (red), and GAD65 pulsed mDCs co-cultured with primed WJ-MSCs (blue). Data were calculated from three different experiments for each patient. Experiments performed in triplicate for each patient. DCs from five patients were used. Statistical significance was tested using a two-way ANOVA with Tukey’s post hoc for multiple comparisons. In all analyses *p < 0.05, ***p < 0.001 and ****p < 0.0001.

The phenotypic analysis of monocyte-generated mDCs cultured in the presence of primed WJ- MSCs showed skewing of mDCs toward an immature state with decreased expression of all co-stimulatory molecules and maturation markers (CD80, CD84, CD86, CD40, CD1a, CD209, and HLA-DR) (**Figure 2**).

### Effect of Primed WJ-MSCs on Activated T Cells Profile and Function

**Figure 3:**
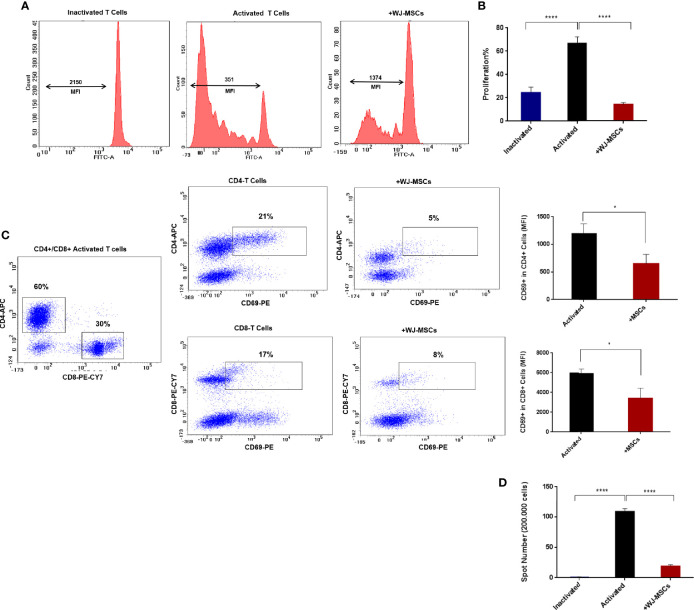
T cells proliferation and activation and IFN-γ ELISPOT analysis **(A)** Representative flow cytometry analysis and mean fluorescent intensity (MFI) the dilution of CFSE fluorescent dye **(B)** Percentage of T cell proliferation under the following conditions: inactivated T cells (blue), activated T cells cultured without (black) and with primed WJ-MSCs (red). Statistical significance was tested using a one-way ANOVA with Tukey’s post hoc for multiple comparisons **(C)** Representative flow cytometry analysis of CD4+/ CD8+ activated T cells and activated CD69+ T cells cultured with and without primed WJ-MSCs and MFI ± SD of CD69+ T cell in CD4+ and CD8+ activated T cells under the following conditions: activated T cells alone (black) and activated T cells co-cultured with primed WJ-MSCs (red). An unpaired *t*-test was performed to test statistical significance **(D)**. Mean ± SD of IFN-γ spots per well (200.000 cells) under the following conditions: inactivated T cells (blue), activated T cells cultured without (black) and with primed WJ-MSCs (red). Statistical significance was tested using a one-way ANOVA with Tukey’s post hoc for multiple comparisons. Data were calculated from three different experiments for each patient. Experiments performed in triplicate for each patient. T cells from five patients were used. For all analyses *p < 0.05, and ****p < 0.0001.

No proliferation was observed in quiescent or inactivated T cells while activated T cells showed high proliferation rate which decreased significantly when co-cultured with primed WJ-MSCs (**Figures 3A, B**). Activated T cells also exhibited a significant decrease in activated CD69+ T cells. Furthermore, the percentage of CD4+ and CD8+ T cells was also significantly decreased from 21% to 5% of CD4+ and from 17% to 8% of CD8+ (**Figure 3C**). Moreover, ELISPOT analysis showed significant inhibition of positive IFN-γ response to GAD65 when activated T cells were co-cultured with primed WJ-MSCs (**Figure 3D**).

### Effect of Primed WJ-MSCs on the Secretion Level of Pro-Inflammatory and Anti-Inflammatory Cytokines

**Figure 4:**
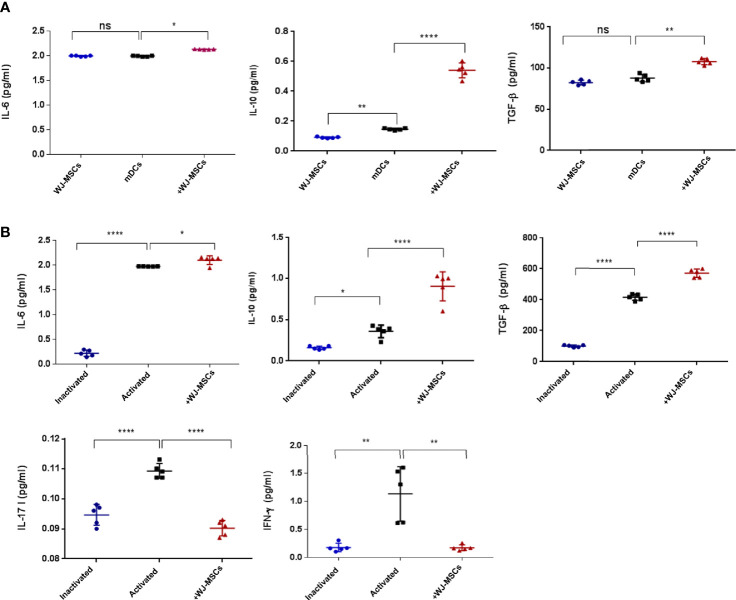
Cytokines secretion level. **(A)** Mean ± SD levels of secreted IL-6, IL-10, and TGF-β in cell free culture supernatant under the following culture conditions: primed WJ-MSCs alone (blue), GAD65- pulsed mDCs co-cultured without (black) and with primed WJ-MSCs (red). **(B)** Mean ± SD levels of secreted IL-6, IL-10, TGF-β, IL-17 and IFN-γ in cell free culture supernatant under the following culture conditions: inactivated T cells (blue), activated T cells without (black) or with primed WJ-MSCs (red). Data were calculated from five patients. Experiments performed in duplicate for each patient. In all analyses *p < 0.05, **p < 0.01 and ****p < 0.0001. ns, not significant.

The secretion levels of anti-inflammatory cytokines (IL-6, IL-10 and TGF-β) were significantly increased when mDCs were co-cultured with primed WJ-MSCs (**Figure 4A**). Furthermore, the secretion levels of anti-inflammatory cytokines (IL-6, IL-10 and TGF-β1) were significantly increased when activated T cells were co-cultured with primed WJ-MSCs. Activated T cells secreted larger amounts of IFN-*γ* and IL-17 than quiescent or inactivated T cells. The levels of IFN-*γ* and IL-17 were significantly decreased when activated T cells were co-cultured with primed WJ-MSCs (**Figure 4B**).

### TNF-α and IFN-γ Priming WJ-MSCs Enhances the Expression of Immunomodulatory Genes and Impair the Expression of Immunostimulatory Genes in mDCs and Activated T Cells

**Figure 5:**
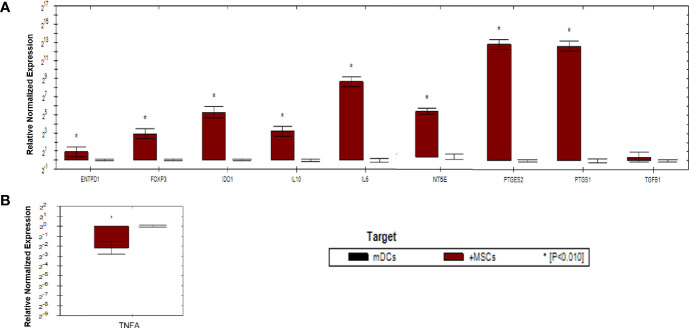
Relative normalized expression of immunomodulatory and immunostimulatory genes of mDCs after co-cultured with primed WJ-MSCs. **(A)** upregulated genes. **(B)** downregulated gene. Mature dendritic cells from four patients were used. Results were normalized to 18S rRNA Each sample was performed in triplicate, and a mean value was calculated. Data were analyzed according to 2−ΔΔCT method using CFX Maestro™ Software - Bio-Rad. *p ≤ 0.01 and fold change ≥ 1.5.

As shown in (**Figure 5**), gene expression profile was evaluated in mDCs. The results showed significant increase in the expression of immunoregulatory genes *(IL10, IDO1, NT5E/ENTPD1, FOXP3, IL6, and PTGES2/PTGS1).* In addition, they showed insignificant increase in the expression of *TGFB1.* Furthermore, mDCs showed significant increase in the expression of *TNFA.* Fold regulation results confirmed the upregulation and downregulation of mDCs target genes. Also, no expression of *IFNG, IL-17A* and, IL*-2* was detected in mDCs (**Table 4**).

**Table 4:** Upregulated and downregulated genes in mDCs after co-cultured with primed WJ-MSCs.

Gene symbol	Fold regulation 2−ΔΔCT	P value
TGFB1	1.3	0.010785
IL10	9	0.000062
IL6	402	0.000028
IDO1	38.4	0.000778
FOXP3	7.5	0.000175
PTGS1	10.2	0.007765
ENTPD1	1.9	0.000871
NT5E	12.4	0.000961
PTGES2	7106	0.000088
IFNG	NA	NA
TNFA	-4.5	0.000562
IL17A	NA	NA
1L2	NA	NA

Results were normalized to 18S rRNA Each sample was performed in triplicate, and a mean value was calculated. Data were analyzed according to 2−ΔΔCT method using CFX Maestro™ Software - Bio-Rad. p ≤ 0.05 and fold change ≥ 1.5. NA, not applicable.

**Figure 6:**
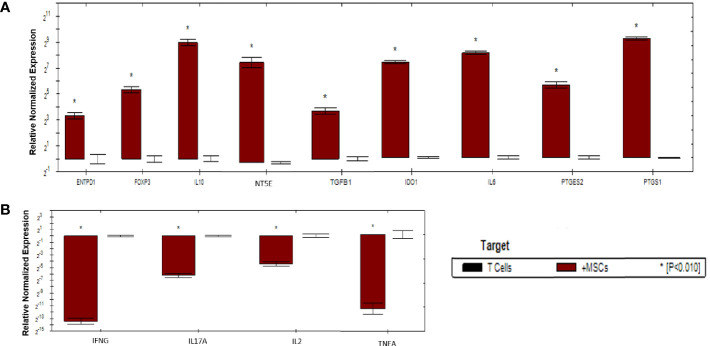
Relative normalized expression of immunomodulatory and immunostimulatory genes of activated T cells after co-cultured with primed WJ-MSCs. **(A)** upregulated genes. **(B)** downregulated genes. T cells from four patients were used. Results were normalized to 18S rRNA Each sample was performed in triplicate, and a mean value was calculated. Data were analyzed according to 2−ΔΔCT method using CFX Maestro™ Software - Bio-Rad. *p ≤ 0.01 and fold change ≥ 1.5.

As shown in (**Figure 6**), activated T cells showed significant increase in the expression of immunoregulatory genes *(IL10, IDO1, NT5E/ENTPD1, FOXP3, IL6, and PTGES2/PTGS1).* Significant increase in the expression of *TGFB1* was detected in activated T cells compared to mDCs. Moreover, significant downregulation of immunostimulatory genes (*TNFA, IFNG, IL-17A*, and *IL-2)* was also detected. Fold regulation results confirmed the upregulation and downregulation of activated T cells target genes. However, T cells exhibited more upregulation and downregulation in gene expression than mDCs (**Table 5**).

**Table 5:** Upregulated and downregulated genes upon priming WJ-MSCs with TNF-α and IFN-γ and cultured with activated T cells.

Gene symbol	Fold regulation 2−ΔΔCT	P value
TGFB1	13	0.000567
IL10	499.1	0.002305
IL6	177.5	0.000057
IDO1	112	0.000089
FOXP3	40.5	0.000541
PTGS1	369	0.000001
ENTPD1	6151.5	0.001495
NT5E	1639.7	0.000197
PTGES2	36	0.002198
IFNG	-11191	0.002797
TNFA	-8.3	0.000545
IL17A	-74.8	0.000117
1L2	-21.1	0.008139

Results were normalized to 18S rRNA Each sample was performed in triplicate, and a mean value was calculated. Data were analyzed according to 2−ΔΔCT method using CFX Maestro™ Software - Bio-Rad. p ≤ 0.05 and fold change ≥ 1.5. NA, not applicable.

## Discussion

Type 1 diabetes is a well-known autoimmune disease characterized by specific adaptive immunity against β-cell antigens. Type 1 diabetes occurs when the balance between the regulatory and inflammatory T-cells is lost. To date, there is no effective therapeutic approach for the management of T1D but recently, MSCs have been reported as a promising immunosuppressant in various autoimmune diseases including T1D (39, 40). However, successful MSCs-based therapy still faces obstacles due to the high sensitivity of MSCs to the environment of immune-mediated diseases, the differences of culturing protocols, and the cell senescence that results from overexpansion of cells. Therefore, a current concern is how to enhance the immunomodulatory effects of MSCs.

In this study, the immunomodulatory effects of primed WJ-MSCs were investigated. The results showed that high differentiation potential and unique morphological features were obtained when WJ-MSCs were primed with both IFN-*γ* and TNF-*α*. Accordingly, our findings provide an additional evidence that the morphological appearance can be used to predict the function of MSCs *in vitro* when pre-conditioned with both *TNF-α and IFN-γ*.

Primed WJ-MSCs exerted an immunomodulatory effect on mDCs by skewing toward tolerogenic or immature phenotype. Tolerogenic DCs express low amounts of co-stimulatory molecules on their surfaces and display increased production of anti-inflammatory cytokines including IL-6, IL-10, and TGF-β1. Furthermore, tolDCs are capable of driving T cells to differentiate into Tregs. This increase is a result of the overexpression of *IL6, IL10*, and *FOXP3.*

Primed WJ-MSCs exhibited immunomodulatory effect on CD4+ and CD8+ T cells by producing tolDCs which inhibit antigen-specific T cell responses through induction T cell anergy.

The levels of IFN-*γ* were significantly decreased when activated T cells were co-cultured with primed WJ-MSCs. This led us to conclude that primed WJ-MSCs suppress T cells-mediated autoimmunity. It is well known that IFN-*γ* are produced extensively by activated T cells and it is a key moderator of T cells-mediated immunity (41, 42).

As for the production of immunomodulatory factors by mDCs and by activated T cells after co-culturing with primed WJ-MSCs, significant upregulation of *IDO1* expression in both mDCs and activated T cells was detected. This provides a potent evidence which confirms that priming WJ-MSC with TNF-*α* and IFN-*γ* promotes the immunosuppressive potential of these cells. This can be considered a critical finding because quiescent MSCs are unable to express *IDO1* (43). However, *IDO1* is considered one of the key modulators of the immune tolerance mechanism (43, 44), and is involved in the tryptophan catabolites or depletion (45–47) which is responsible for the inhibition of T cell activation and proliferation *via* the induction of T cell anergy (48, 49).

Both TGF-β1 and IL-10 are critical anti-inflammatory cytokines for Treg formation (50, 51). Moreover, they are capable of inhibiting T cells proliferation and activation as well as suppressing of Th17 generation. These results are consequences of the increase in the production of TGF-β1, IL-10, IFN-γ, and IL-17 after activated T cells are co-cultured with primed WJ-MSCs. Furthermore, TGF-β1 is a key anti-inflammatory cytokine which is responsible for the formation of Tregs due to its ability to upregulate *FOXP3*.

The downregulation of *IFNG, IL2* and IL*17A* expression and the significant reduction of IFN-γ and IL-17 cytokines after activated T cells were co-cultured with primed WJ-MSCs represent a strong evidence of the suppression of T cells proliferation and activation. In addition, the downregulation of *IL2* represents a strong evidence of the inhibition of T cells-mediated autoimmunity because *IL2* are produced only by CD4+ and CD8+ T cells. Moreover, IL2 plays a pivotal role in T cells- receptor signaling pathway (52).

Significant expression of *PTGS1* and *PTGES2* was observed when primed WJ-MSCs were co-cultured with mDCs and activated T cells. *PTGES2* and *PTGS1* genes are responsible for the production of prostaglandin E2 (PGE2) which is involved in the immune-suppressive mechanism of MSCs and it increases the expression of anti-inflammatory factors (53–55). The upregulation of *IL10*, and *TGFB1* as well as the significant production of TGF-β1 and IL-10 might be explained by the overexpression of PTGS1 and PTGES2.

Interleukine-6 (IL-6) is a key factor in the formation of Tregs and in the suppression of pro-inflammatory responses (56, 57). The upregulation of *IL6* gene and the increase of IL-6 cytokine may explain the inhibition of pro-inflammatory responses of mDCs and activated T cells which is caused by primed WJ-MSCs. Moreover, a previous study illustrated that immunosuppressive properties of amnion-derived MSCs are not constitutive, but require a supportive signal to produce PGE2, IDO1, and a high level of IL-6 (51). Our findings may support the immunomodulatory effect of primed WJ-MSCs on mDCs and activated Tcells.

Destructed β cells play a key role in the progression of T1D through the release of dangerous extracellular ATP signal which acts as a potent immune-stimulator to enhance inflammatory responses. So, we investigated the effect of primed WJ-MSCs on the expression of *ENTPD1/NT5E* genes which play a pivotal role in the production of extracellular adenosine through ATP hydrolysis. However, extracellular adenosine act as a potent immune-regulator signal that modulate innate and adaptive immunity. Moreover, adenosine can prevent the activation, proliferation and cytokine production in CD4+ and CD8+ T cells (58–60). ENTPD1/NT5E are considered the most important genes in the immunosuppressive mechanisms that attribute to Treg formation and T cell anergy (61, 62). Furthermore, they maintain the balance between ATP/adenosine to increase immune hemostasis (63). Significant increase in the expression of *ENTPD1/NT5E* may be considered a robust finding because of their role in maintaining balance between anti-inflammatory and pro-inflammatory factors.

Although the direct contact with MSCs is critical for the immunomodulatory effects, the paracrine effects of MSCs cannot be ignored. The Increase in the production of anti-inflammatory cytokines (IL-10, IL-6 and TGF-β1) and the suppression in the production of pro-inflammatory cytokines (IL-17 and IFN-γ) in conditioned media suggest that the immunomodulatory effects of primed WJ-MSCs after being co-cultured with mDC and T cells seems to be a consequence of a synergic effect mediated by both the direct contact and secretome of primed WJ-MSCs.

Collectively, this study confirmed that WJ-MSCs primed by IFN-γ and TNF-α modulated mDCs-mediated antigen presentation through the induction of tolDCs in addition to the modulation of antigen-specific-T cell responses through the induction of T cell anergy. More importantly, this study paves the road to utilizing primed WJ-MSCs-based transplantation therapies. Further study of primed WJ-MSCs cellular therapies in an animal model of T1D is recommended before attempting in humans. Successful treatment in humans would involve establishing safety first, then optimizing administration in respect to disease stage. Premature of primed WJ-MSCs therapies in human would not only risk safety and efficacy, but also provide false hope to patients.

## Data Availability Statement

The raw data supporting the conclusions of this article will be made available by the authors, without undue reservation.

## Ethics Statement

The protocol was approved by Institutional Review Board (IRB) committee of the Cell Therapy Center, The University of Jordan, Jordan (IRB NO. 07-11-2019). Written informed consent to participate in this study was provided by the participants’ legal guardian/next of kin.

## Author Contributions

MA-M designed and conducted the study. MA-M and SM analyzed and interpreted data. MA-M wrote the original manuscript. NA revised the manuscript. HJ and AA were the administrators of the project. AA provided the advices for study. All authors contributed to the article and approved the submitted version.

## Funding

This work was financed by The University of Jordan, Dean of Scientific Research, and Cell Therapy Center/The University of Jordan.

## Conflict of Interest

The authors declare that the research was conducted in the absence of any commercial or financial relationships that could be construed as a potential conflict of interest.

## Publisher’s Note

All claims expressed in this article are solely those of the authors and do not necessarily represent those of their affiliated organizations, or those of the publisher, the editors and the reviewers. Any product that may be evaluated in this article, or claim that may be made by its manufacturer, is not guaranteed or endorsed by the publisher.
